# Fitness Profile of Police Officers from Rapid Intervention Teams of the Lisbon Metropolitan Command

**DOI:** 10.3390/jfmk10010090

**Published:** 2025-03-11

**Authors:** João Daniel Freitas, Luís Miguel Massuça

**Affiliations:** 1 Higher Institute of Police Sciences and Internal Security, 1300-663 Lisbon, Portugal; jfreitas@psp.pt; 2 ICPOL—Police Research Centre, Higher Institute of Police Sciences and Internal Security, 1300-663 Lisbon, Portugal; 3 CIDEFES, Lusófona University, 1749-024 Lisbon, Portugal; 4 CIFI2D, Faculty of Sport, University of Porto, 4200-450 Porto, Portugal

**Keywords:** age, law enforcement, physical tests, physical training, security

## Abstract

**Background**: A rapid intervention team is a broad category of special teams used by police and emergency respondents to cover various needs. It is essential to ensure the safety and well-being of people in emergencies, minimising the risk of harm and maximising the chances of survival. **Objective**: This study aimed (i) to identify the fitness profiles and levels of POs from the EIR of the Lisbon Metropolitan Command (COMETLIS, PSP, Portugal), considering age classes; (ii) to directly compare the observed fitness profiles to previous research and normative data; and (iii) to compare the fitness profile of POs from the EIR with cadets from the Police Academy. **Methods**: This cross-sectional observational study included the participation of 121 male POs from the EIR of the Lisbon Metropolitan Command (Portugal) and 92 male cadets from the Police Academy (Lisbon, Portugal). The assessment protocol sequence involved the collection of biosocial data (age classes: ≤29 years; 30–39 years; 40–49 years), a body size assessment, and a fitness assessment (horizontal jump, handgrip strength, 60 s sit-ups and 20 m shuttle run). **Results**: (i) In the ≤29 years age class, POs performed better in all fitness tests (highlighting that the age class had a statistically significant effect on performance in the horizontal jump, sit-ups, 20 m shuttle run, and predicted *V*O_2_max), and they showed significantly better performance than cadets in handgrip (left, right, and sum), and significantly worse performance in sit-ups and predicted *V*O_2_max. (ii) In the 30–39 years age class, POs had significantly worse performance than cadets in the horizontal jump, sit-ups, 20 m shuttle run, and predicted *V*O_2_max, even after controlling for age. **Conclusions**: (i) The fitness performance decreased as the age class became older; (ii) the handgrip strength and cardiovascular capacity attributes were between the standard and excellent levels according to the ACSM guidelines for the general population; (iii) POs from the EIR were stronger than cadets in terms of handgrip strength but weaker in terms of lower limb power, abdominal muscular endurance, and aerobic capacity; and (iv) the differences observed between POs from the EIR and cadets in the 30–39 years age class emphasise the importance of physical training after the training period and throughout professional life.

## 1. Introduction

Levels of intervention are used to organise the work of the Portuguese Public Security Police (PSP), and its police officers (POs) must have adequate fitness profiles to fulfil their mission and perform their duties. As the level of intervention required to respond to various incidents increases (i.e., higher levels), the level of response requires that the POs be more physically prepared (since the tasks are more demanding) so as not to endanger their safety and that of the population [1].

To assess the fitness profile of POs, there are fitness tests for admission to the various progression and specialisation courses to ensure that these POs have the necessary skills to complete the courses and perform their future duties. Due to their specific responsibilities, POs in the Special Police Unit (the last intervention level of the PSP) must pass annual fitness certification tests to maintain and/or renew their service commission.

POs from the rapid intervention teams (EIRs) of the Lisbon Metropolitan Command (COMETLIS, PSP, Portugal), included in the intermediate intervention level of the PSP, must complete the Police Intervention Techniques Course to perform their duties. According to Fernandes [2], fitness tests should be one of the requirements for access to the Police Intervention Techniques Course (some COMETLIS Police Divisions already present this as a requirement for the approval of fitness tests).

Already present as part of POs’ initial training to prepare and develop the body for the physical tasks required of POs [3], physical instruction time becomes essential not only during courses (through physical education hours) but also throughout their police careers to avoid situations of stress and anxiety or even reduce the risk of injuries [4].

POs need specific physical training programs to perform their tasks, which should be maintained throughout their professional careers [5]. The PSP provides physical training time for police officers from EIR to maintain/increase their fitness levels. This was recently observed by Massuça and Rasteiro [6], who, after implementing a 12-week physical exercise program to develop the individual fitness attributes of each PO, observed significant improvements in all fitness attributes, i.e., improvements in the PO fitness profile.

A consensus is that fitness attributes are essential for POs’ duties, performance, and general health. Previous studies identified the most used tests of POs’ physical fitness and health evaluations and documented, compared, and examined the reference values available in the current literature regarding the most used fitness tests to assess and predict PO performance [7,8]. Although the fitness profile of several police populations has been the subject of study, there is no information in the literature about the fitness profile of this specific tactical population of POs from the EIR of the Lisbon Metropolitan Command (Portugal).

In accordance, this study aimed (i) to identify the fitness profiles and levels of POs from the EIR of the Lisbon Metropolitan Command (COMETLIS, PSP, Portugal), considering age classes; (ii) to directly compare the observed fitness profile to previous research and normative data published in the American College of Sports Medicine (ACSM) guidelines; and (iii) to compare the fitness profile of POs from the EIR with cadets from the Police Academy. We hypothesise that the fitness level of POs from the EIR (i) decreases with increasing age class, (ii) is very good in all age classes according to previous research and normative data, and (ii) is similar to that of cadets from the Police Academy.

## 2. Materials and Methods

### 2.1. Research Design

In this observational cross-sectional study, a fitness assessment protocol for POs [9] was applied to POs from the EIR of the Lisbon Metropolitan Command (COMETLIS, PSP, Portugal) and cadets from the Police Academy (i) to identify the fitness profile and level of POs from EIR, and (ii) to confirm the effect of age classes on fitness profile. Complementarily, the identified fitness profile was compared (i) to previous research and normative data and (ii) with the fitness profile of cadets from the Police Academy. In accordance, the fitness assessment protocol was applied (in a single session) in the following order: (i) briefing, (ii) acceptance of informed consent, (iii) collection of biosocial data, (iv) anthropometric assessment, and (v) fitness assessment.

### 2.2. Participants

A total of 121 male POs from the EIR (age groups: ≤29 years, n = 52; 30–39 years, n = 60; 40–49 years, n = 9) belonging to the COMETLIS police divisions (participation rate of ~30%), and 92 male cadets (age groups: ≤29 years, n = 66; 30–39 years, n = 26) from the Police Academy (Higher Institute of Police Sciences and Internal Security—ISCPSI, Lisbon, Portugal) participated in this study (Table 1), with all participants being informed of the objectives of the investigation and agreeing to participate (through informed consent) before evaluations. The Portuguese PSP and the Portuguese Police Academy (ISCPSI) approved this study (approval code: 285/SECDE/2023; approval date: 29 November 2023).

### 2.3. Procedures and Instruments

To assess the biosocial characteristics of the participants, the following age classes were considered: ≤29 years, 30 to 39 years, and 40 to 49 years).

The anthropometric assessment consisted of two anthropometric measurements: (i) body height (m) and (ii) body mass (kg). A measuring tape wall was used to measure height (Holtain Ltd., Crymych, UK) with the smallest scale of 0.1 cm, and body mass was obtained using a digital scale (TANITA, Dual Frequency Body Composition Monitor, RD-953-bk, Tanita Ltd., Amsterdam, The Netherlands). In both measurements, participants were barefoot and dressed only in shorts and a t-shirt.

**Figure 1:**
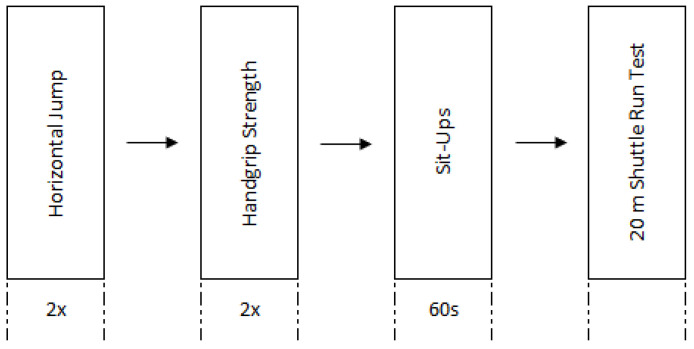
Fitness assessment protocol scheme.

The fitness assessment protocol comprised the following fitness tests: (i) horizontal jump (HJ); (ii) handgrip strength (HG); (iii) 60 s sit-ups; and (iv) 20 m shuttle run (Figure 1. The protocol was applied on the same day, with similar weather conditions, and the variables under analysis were (i) left HG (kg); (ii) right HG (kg); (iii) HG sum (kg); (iv) HJ (m); (v) 60 s sit-ups (n); (vi) 20 m shuttle run test (n); and (vii) *V*O_2_max (mL/kg/min). All participants completed a familiarisation session, and the order of the fitness tests (Figure 1) assumed a progressive increase in fatigue [6], with a ten-minute interval between tests. The fitness assessment protocol for POs was recently published, and a detailed description can be seen in Freitas et al. [9].

### 2.4. Statistical Analysis

Descriptive statistics are presented through central tendency (mean) and dispersion (standard deviation) measures. The analysis of variance (ANOVA) was used, followed by the post hoc Bonferroni test, to analyse whether age class (≤29 years; 30–39 years; 40–49 years) significantly influenced the fitness performance of the male POs. The ANOVA was also used to analyse, in the ≤29 years and 30–39 years age groups, the differences in fitness performance between POs and cadets. In continuation, the effect of these operational groups on fitness performance was also evaluated, controlling for the effects of chronological age (ANCOVA, covariant: age). The effect size was expressed using eta squared (η^2^) and was interpreted as follows: >0.01, small; 0.06, medium; >0.138, large [10]. All statistical analyses and graphical representations were performed using the JASP computer program (JASP 0.18.3, JASP Team, 2024) [11].

## 3. Results

POs aged ≤29 years performed better in all fitness tests, highlighting that the age class had a statistically significant effect on performance in the horizontal jump (*F*(2,118) = 12.192, *p* < 0.001; ES, large), 60 s sit-ups (*F*(2,118) = 4.047, *p* < 0.05; ES, moderate), 20 m shuttle run (*F*(2,118) = 4.118, *p* < 0.05; ES, moderate), and predicted *V*O_2_max (*F*(2,118) = 3.761, *p* < 0.05; ES, moderate). In addition, according to the multiple comparisons, it was observed that the age class ≤29 years showed (i) significantly higher performance in the horizontal jump than POs in the 30–39 years (*p* < 0.001) and 40–49 years age classes (*p* < 0.01); (ii) significantly higher performance in 60 s sit-ups than POs in the 40–49 years age class (*p* < 0.05); (iii), significantly higher performance in the shuttle run test (n) and predicted *V*O_2_max than POs in the 30–39 years age class (both *p* < 0.05). The results are presented in Table 2.

Comparing POs with cadets, it was observed that POs in the ≤29 years age class showed (i) significantly better performance in left handgrip (*F*(1,116) = 15.595, *p* < 0.001; ES, moderate), right handgrip (*F*(1,116) = 18.722, *p* < 0.001; ES, large), and handgrip sum (*F*(1,116) = 18.322, *p* < 0.001; ES, moderate), and (ii) significantly worse performance in 60 s sit-ups (*F*(1,116) = 13.302, *p* < 0.001; ES, moderate) and predicted *V*O_2_max (*F*(1,116) = 7.644, *p* < 0.01; ES, moderate). In addition, the ANCOVA (covariant: age) results only revealed significant differences in sit-ups (*F*(1,116) = 7.611, *p* < 0.01; ES, moderate).

**Figure 2:**
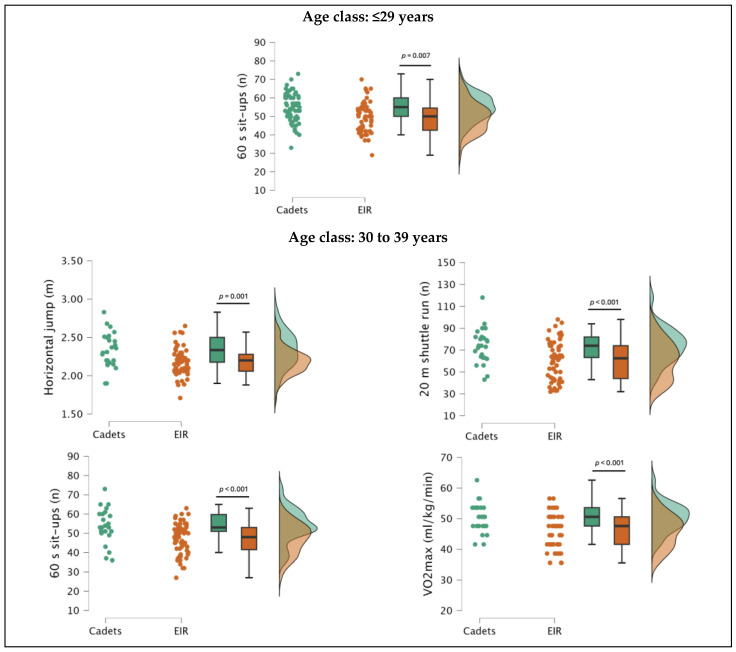
Distribution and differences in fitness attributes of male police officers (POs) from the Lisbon Met-ropolitan Command (Portugal) rapid intervention teams (EIR) and cadets from the Police Academy, from the ≤29 years and 30–39 years age groups, after controlling for age.

In the 30–39 years age class, POs showed significantly worse performance in the horizontal jump (*F*(1,84) = 11.147, *p* < 0.01; ES, moderate), 60 s sit-ups (*F*(1,84) = 12.275, *p* < 0.001; ES, moderate), 20 m shuttle run (*F*(1,84) = 11.299, *p* < 0.01; ES, moderate), and predicted *V*O_2_max (*F*(1,84) = 14.138, *p* < 0.001; ES, large). In addition, the ANCOVA (covariant: age) results revealed significant differences in the same fitness attributes (horizontal jump, *F*(1,83) = 11.466, *p* < 0.01, ES, moderate; 60 s sit-ups, *F*(1,83) = 12.898, *p* < 0.001, ES, moderate, 20 m shuttle run, *F*(1,83) = 11.695, *p* < 0.001, ES, moderate; predicted *V*O_2_max, *F*(1,83) = 14.728, *p* < 0.001, ES, large). The results are presented in Table 3 and Figure 2.

## 4. Discussion

The application of the fitness assessment protocol to operational PSP POs from the EIR of the Lisbon Metropolitan Command (Portugal) made it possible to understand the levels of the four most studied fitness attributes in POs (i.e., handgrip strength, lower limb muscle power, abdominal muscle resistance, and aerobic capacity).

It was observed that the POs had superior fitness performances to those found in the literature, i.e., (i) in the horizontal jump, they showed improvements of +0.32 m and +0.06 m in comparison to the results of Marins et al. [1] (1.92 ± 0.14 m) and Massuça and Rasteiro [6] (2.18 ± 0.20 m), respectively; (ii) their sum handgrip strength showed an improvement of +17.97 kg compared to that observed in the study by Massuça and Rasteiro [6] (88.77 ± 7.7 kg); (iii) in the 60 s sit-up test, they showed +6.45, +7.33, and +6.70 repetitions in comparison to the results observed by Dawes et al. [12] (41.05 ± 6.96 repetitions), Orr et al. [13] (40.17 ± 7.69 repetitions), and Massuça and Rasteiro [6] (40.80 ± 7.53 repetitions), respectively; and (iv) in the 20 m shuttle run test, they showed an average of 64.90 ± 90 shuttles with an additionally improved predicted *V*O_2_max (+22 mL/kg/min) compared that achieved in the investigation by Massuça and Rasteiro [6] (*V*O_2_max, 42.70 ± 3.74 mL/kg/min).

Considering the studied POs’ age classes (≤29 years, 30–39 years, and 40–49 years), it was observed that POs aged ≤29 years performed better in all fitness tests, highlighting the statistically significant effect of age class in the horizontal jump (higher than the 30–39 years and 40–49 years age classes), sit-ups (higher than the 40–49 years age class), 20 m shuttle run, and predicted *V*O_2_max (higher than the 30–39 years age class). It is also apparent in the literature that the ≤29 years age group achieved superior performances in the handgrip (sum) strength [14], horizontal jump [14], 60 s sit-ups [15,16], and 20 m shuttle run tests [15].

In continuation, it was observed that POs aged ≤29 years presented lower values in the handgrip (sum) strength test (−6.16 kg) in comparison with the results reported by Teixeira et al. [14] (114.34 ± 12.04 kg). However, they presented better fitness performance in (i) the horizontal jump (+0.11 m) compared to Teixeira et al. [14] (2.22 ± 0.15 m); (ii) 60 s sit-ups (+8.32 and +9.93 repetitions, respectively) compared to Dawes et al. [15] (41.17 ± 8.22 repetitions) and Lockie et al. [16] (39.56 ± 7.56 repetitions); and (iii) 20 m shuttle run (+15.08 shuttles) compared to Dawes et al. [15] (55.63 ± 20.90 shuttles).

In the 30–39 years age group, POs obtained better results in all fitness tests, i.e., (i) in the handgrip (sum) strength (+1.91 kg) and horizontal jump (+0.11 m), compared to the study by Teixeira et al. [14] (handgrip sum, 104.79 ± 13.47 kg; horizontal jump, 2.08 ± 0.11 m); (ii) in 60 s sit-ups (+10.49 and +9.65 repetitions, respectively) compared to the investigations by Dawes et al. [15] (36.63 ± 9.67 repetitions) and Lockie et al. [16] (37.47 ± 8.43 repetitions); and (iii) in the 20 m shuttle run (+18.64 shuttles) compared to Dawes et al. [15] (42.19 ± 19.85 shuttles).

Finally, in the 40–49 years age group, and similar to what was observed in the <29 years age group, POs presented inferior fitness performance in the handgrip (sum) strength test (−0.52 kg) than that reported by Teixeira et al. [14] (106.63 ± 15.12 kg). In the remaining studied fitness attributes, they achieved superior performances, i.e., (i) +0.14 m in the horizontal jump compared to Teixeira et al. [14] (1.95 ± 0.17 m); (ii) +9.16 and +6.24 repetitions in 60 s sit-ups compared with Dawes et al. [15] (31.73 ± 9.94 repetitions) and Lockie et al. [16] (34.65 ± 8.40 repetitions), respectively; and (iii) +31.47 shuttles in the 20 m shuttle run test compared with Dawes et al. [15] (31.31 ± 15.52 shuttles).

Complementarily, it also seems crucial to compare the fitness profiles of the studied POs from EIR with the normative data published in the ACSM guidelines for the general population [17]. In accordance, the direct comparison of essential components for health (prevention and promotion), such as handgrip strength and cardiovascular capacity [18], revealed that (i) the handgrip (sum) strength of POs aged ≤29 years (108.18 kg), 30–39 years (106.70 kg), and 40–49 years (97.11 kg) were at the very good level (≤29 years and 30–39 years, 104–114 kg; 40–49 years, 97–107 kg); and (ii) the predicted *V*O_2_max of POs aged ≤29 years (48.70 mL/kg/min) was at the standard level (50th percentile, 48 mL/kg/min [17]), that in the 30–39 years age group (45.90 mL/kg/min) was at the good level (60th percentile, 45.20 mL/kg/min [17]), and that in the 40–49 years age group (46.60 mL/kg/min) was at the excellent level (80th percentile, 46.70 mL/kg/min [17]).

In continuation, when comparing the fitness of male operational service POs from the EIR and male cadets (POs in training) who completed the same fitness protocol, it seems relevant to cross-reference the results to understand this topic better in these populations, i.e., that (i) POs showed stronger handgrip strength, and (ii) cadets showed superior lower limb power, abdominal muscular endurance, and aerobic capacity.

Considering the ≤29 years age group, (i) POs performed significantly better in handgrip strength (left, +5.57 kg; right, +6.57 kg; sum, +12.14 kg) than cadets, but (ii) cadets achieved significantly superior performance in abdominal muscular endurance (+5.39 repetitions) and aerobic capacity (+2.77 mL/kg/min). However, when controlling for age, the observed differences remained statistically significant only in sit-ups, suggesting that the participants’ chronological age could explain the original differences.

Also, in the 30–39 years age group, POs continued to show higher values in the handgrip strength test (left, 0.93 kg; right, 0.25 kg; sum, 1.18 kg), but unlike the ≤29 years age group, they were no longer statistically significant. The trend continued in the remaining fitness attributes, as cadets demonstrated significantly superior performance in the horizontal jump (+0.15 m), abdominal muscular endurance (sit-ups, +8.80 repetitions), and aerobic capacity (20 m shuttle run test, +13.63 shuttles; predicted *V*O_2_max, +4.59 mL/kg/min), even after controlling for chronological age.

From a practical perspective, this observation is meaningful as POs need to complete tasks regardless of their age and rank [19,20]. However, from a scientific perspective, this comparison showed that the difference is not solely due to age differences. The impact of chronological age (covariant) on these comparisons was partially observed in the ≤29 years age group but not in the 30–39 years age group, suggesting that the observed differences may be due to training.

Therefore, it is essential to highlight that the working hours of the POs from the EIR include time for physical training [6] and that the cadets are in the process of academic and operational training (generally, in boarding school), providing general and specific physical fitness. Accordingly, the observed differences suggest that PO fitness is optimised for this function, since handgrip strength was the attribute that (i) showed a superior performance in the two age classes studied, and (ii) even controlling for age, it did not differ significantly from cadets in training. Regarding the other fitness tests, the differences observed between POs from the EIR and the cadets (even after controlling for chronological age), mainly in the 30–39 years age class, suggest that they may be a consequence of the training methodology used in these two contexts, which requires further in-depth study with the aim of clarifying what motivates the observed differences.

Lastly, it should be noted that this study presents some weaknesses, namely the fact that the population studied was specific (male POs from EIR aged ≤ 49 years) and the methodology involved the assessment of a restricted fitness profile. It is suggested that future studies include (i) female participants, (ii) an assessment protocol that considers an assessment of morphological profile, and (iii) more operational groups of the Portuguese public security police (cadets from the Higher Institute of Police Sciences and Internal Security, cadets from the practical police school; POs; POs from the EIR; POs from the Special Police Unit, etc.), to have a broader view of the PO population.

## 5. Conclusions

Understanding potential areas of opportunity for improved fitness and performance of these POs is essential and directly impacts public safety. This research paper represents an important segment and starting point for defining normative values and the fitness profile of POs from the EIR of the Lisbon Metropolitan Command.

In summary, taking into account the fitness attributes investigated in male POs from the EIR of the Lisbon Metropolitan Command (COMETLIS, PSP, Portugal), it seems relevant to highlight that (i) the fitness performance decreased as the age class increased (≤29 years, 30–39 years, and 40–49 years); (ii) the handgrip strength and cardiovascular capacity attributes were between the standard and excellent levels according to the ACSM guidelines for the general population (depending on age class); (iii) POs from the EIR were stronger than cadets from the Police Academy in terms of handgrip strength, but were weaker in lower limb power, abdominal muscular endurance, and aerobic capacity; and (iv) the differences observed between POs from the EIR and cadets in the 30–39 years age class (even after controlling for chronological age) emphasise the importance of physical training after the training period and throughout professional life.

## Figures and Tables

**Table 1:** Participants’ age and body size (mean ± standard deviation).

	Police Officers from “EIR”	Cadets from Police Academy
Age (years)	30.94 ± 5.78	24.49 ± 6.14
Height (m)	1.78 ± 0.07	1.77 ± 0.06
Body mass (kg)	84.28 ± 11.26	75.44 ± 8.38
Years of experience	6.49 ± 5.31	-

**Table 2:** Fitness attributes (mean ± standard deviation) and differences between age groups (≤29 years; 30–39 years; 40–49 years) of male police officers from the Lisbon Metropolitan Command (Portugal) rapid intervention teams (EIR).

Variables	Total	Age Classes			ANOVA			Bonferroni	
		≤29 Years (1)	30–39 Years (2)	40–49 Years (3)	*F*(2,118)	*p*-Value	η^2^	1–2	1–3
Left handgrip (kg)	52.08 ± 7.34	52.90 ± 8.56	52.14 ± 6.20	46.93 ± 4.97	2.602	0.078	0.042		
Right handgrip (kg)	54.55 ± 8.01	55.28 ± 9.36	54.56 ± 6.86	50.18 ± 5.65	1.575	0.211	0.026		
Handgrip sum (kg)	106.62 ± 14.76	108.18 ± 17.47	106.70 ± 12.39	97.11 ± 8.53	2.203	0.115	0.036		
Horizontal jump (m)	2.24 ± 0.20	2.33 ± 0.20	2.19 ± 0.18	2.09 ± 0.16	12.192	<0.001	0.171	<0.001	0.001
60 s sit-ups (n)	47.66 ± 8.83	49.49 ± 8.41	47.12 ± 8.07	40.89 ± 12.73	4.047	0.020	0.065		0.020
20 m shuttle run (n)	65.22 ± 18.83	70.71 ± 18.28	60.83 ± 17.66	62.78 ± 23.25	4.118	0.019	0.065	0.016	
*V*O_2_max (mL/kg/min)	47.15 ± 5.54	48.70 ± 5.28	45.90 ± 5.37	46.60 ± 6.54	3.761	0.026	0.060	0.022	

**Table 3:** Fitness attributes of cadets from the Police Academy; mean differences between male police officers (POs) from the Lisbon Metropolitan Command (Portugal) rapid intervention teams (EIR) and cadets, ANOVA and ANCOVA (covariant: age) results, considering age groups ≤29 years and 30–39 years.

Variables	Cadets	EIR—Cadets	ANOVA			ANCOVA (Covariant: Age)		
Age Class: ≤29 Years	Mean ± SD	Mean Difference (95% CI)	*F*(1,116)	*p*-Value	η^2^	*F*(1,115)	*p*-Value	η^2^
Left handgrip (kg)	47.32 ± 6.78	5.573 (2.778 to 8.369)	15.595	<0.001	0.119	2.838	0.095	0.021
Right handgrip (kg)	48.72 ± 7.13	6.565 (3.560 to 9.570)	18.722	<0.001	0.139	3.137	0.079	0.023
Handgrip sum (kg)	96.04 ± 13.34	12.138 (6.522 to 17.755)	18.322	<0.001	0.136	3.195	0.076	0.023
Horizontal jump (m)	2.38 ± 0.15	−0.042 (−0.104 to 0.021)	1.748	0.189	0.015	0.462	0.498	0.004
60 s sit-ups (n)	54.88 ± 7.53	−5.389 (−8.315 to −2.462)	13.302	<0.001	0.104	7.611	0.007	0.060
20 m shuttle run (n)	77.61 ± 19.32	−6.895 (−13.825 to 0.036)	3.883	0.051	0.032	0.861	0.355	0.007
*V*O_2_max (mL/kg/min)	51.46 ± 5.49	−2.767 (−4.750 to −0.785)	7.644	0.007	0.062	1.669	0.199	0.014
Age class: 30–39 years	Mean ± SD	Mean difference (95% CI)	*F*(1,84)	*p*-value	η^2^	*F*(1,83)	*p*-value	η^2^
Left handgrip (kg)	51.21 ± 7.69	0.930 (−2.187 to 4.048)	0.352	0.555	0.004	0.380	0.540	0.004
Right handgrip (kg)	54.31 ± 8.42	0.254 (−3.183 to 3.691)	0.022	0.884	2.571 × 10^−4^	0.028	0.869	3.275 × 10^−4^
Handgrip sum (kg)	105.52 ± 15.23	1.184 (−5.024 to 7.392)	0.144	0.705	0.002	0.161	0.689	0.002
Horizontal jump (m)	2.34 ± 0.23	−0.152 (−0.242 to −0.061)	11.147	0.001	0.117	11.466	0.001	0.119
60 s sit-ups (n)	53.92 ± 8.66	−6.804 (−10.667 to −2.942)	12.275	<0.001	0.129	12.898	<0.001	0.131
20 m shuttle run (n)	74.46 ± 16.30	−13.628 (−21.691 to −5.566)	11.299	0.001	0.119	11.695	<0.001	0.121
*V*O_2_max (mL/kg/min)	50.48 ± 4.76	−4.585 (−7.009 to −2.160)	14.138	<0.001	0.144	14.728	<0.001	0.146

## Data Availability

The data presented in this study are available upon reasonable request from the corresponding author. The data are not publicly available due to privacy and ethical restrictions.
